# Knee extension contracture with fixed anterior tibia subluxation treated with PCL release and quadricepsplasty: a case report

**DOI:** 10.1186/s40634-023-00703-y

**Published:** 2023-11-29

**Authors:** Dong Woon Kim, Marcin Mostowy, Przemyslaw A. Pękala, Michalina Bawor, Nicholas I. Kennedy, Robert F. LaPrade, Konrad Malinowski

**Affiliations:** 1 Department of Anatomy, Jagiellonian University Medical College, Kraków, Poland; 2 Artromedical Orthopaedic Clinic, Antracytowa 1, 97-400 Bełchatów, Poland; 3 Orthopedic and Trauma Department, Veterans Memorial Teaching Hospital in Lodz, Medical University of Lodz, 90-549 Lodz, Poland; 4 Faculty of Medicine and Health Sciences, Andrzej Frycz Modrzewski Kraków University, Kraków, Poland; 5 Medical University of Lodz, Lodz, Poland; 6 Twin Cities Orthopedics, 4010 W 65Th St Edina, Minnesota, 55435 USA

**Keywords:** Posterior cruciate ligament, Release, Severe, Extension contracture, Flexion deficit, fixed anterior tibial subluxation, Quadricepsplasty

## Abstract

58-year-old male presented with knee extension contracture (25°) with iatrogenic fixed anterior tibial subluxation. Consecutive arthroscopic arthrolysis, manipulation under anesthesia, and quadriceps-Z-plasty during one surgery failed to restore flexion. Therefore, shortened posterior cruciate ligament was released, which eliminated subluxation and allowed 115° flexion. Despite physiotherapy, flexion progressively decreased to 70° postoperatively. Revision quadricepsplasty by transverse incisions restored 120° of flexion maintained at 31-months follow-up. International Knee Documentation Committee increased 4/87- > 50/87, Knee injury and Osteoarthritis Outcome 7/100- > 68/100 at follow-up. Posterior cruciate ligament release and repeated quadricepsplasty could be a viable salvage option in severe extension contracture with fixed anterior tibial subluxation.

## Introduction

Mild knee flexion deficits may be well tolerated. However, severe extension contracture can have a devastating impact both on a patient’s quality of life and knee joint itself. In most cases, extensive arthroscopic arthrolysis of intra-articular adhesions, accompanied with manipulation under anesthesia (MUA), is sufficient to restore knee flexion. However, in cases of greater extension contracture, procedures involving tendon or ligament may be necessary [1, 2]. What is more, the role of fixed tibial subluxation with incorrect joint congruence and subsequent “hinge-like” knee movement has not been well-described in literature to authors’ knowledge [3].

This paper aims to describe a case report of severe knee extension contracture with fixed anterior tibial subluxation, resulting in incorrect congruence of the joint surfaces refractory to standard procedures.

### Case report

**Fig. 1:**
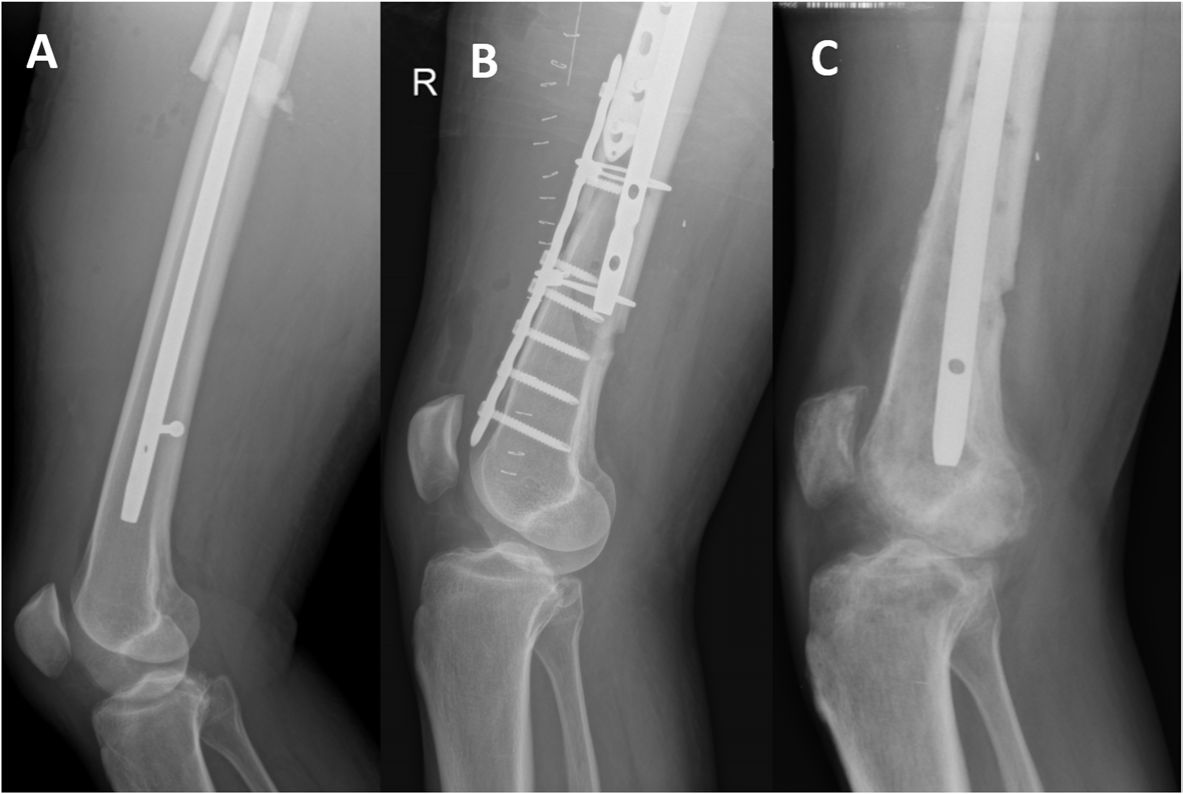
**A** X-ray seven days after initial open reduction and intramedullary nailing (nail Gamma Long, Medgal), June 2017. Destabilization of the fixation and migration of the femoral fragments can be seen. Anterior tibial subluxation is not present yet. **B** Postoperative X-ray after first reoperation due to destabilization of initial fixation, June 2017. Anterior tibial subluxation can be seen. **C** Postoperative X-ray after second reoperation due to nonunion, April 2018. Anterior tibial subluxation and limited knee flexion is still present despite manipulation under anesthesia

The 58-year old male patient sustained a crushing injury to the right thigh in June 2017. As the result of the accident, open (Gustilo Grade I) multifragmentary fracture of the femoral shaft and neck occurred. What is more, there was massive direct injury to anterior proximal half of thigh muscles and soft tissues. However, there was no injury to the knee at the time of accident and full stability of the knee was maintained at this point, with no subluxation in any direction. He was treated with open reduction and intramedullary nailing (Long Gamma Nail, Medgal). After this procedure, more than 90 degrees of passive knee flexion was possible without knee pain and full knee extension was possible. Seven days later, he was re-operated due to destabilization of the fixation and migration of the femoral fragments (Fig. 1A). Normal joint congruence was still present, with no tibial subluxation visible on X-ray. During the procedure, an intraoperative, iatrogenic fracture of distal femur occurred. The fractures after reduction were further stabilized with the use of two LCP plates (company not reported) and four cerclage cables (Stryker). Distal stabilization screw of the intramedullary nail was removed. On postoperative radiographs, good fracture reduction was demonstrated, however, anterior tibial subluxation could already be noticed (Fig. 1B). While there was no available report of the status of the knee ligaments at this time, such anterior tibial subluxation was clearly associated with anterior cruciate ligament (ACL) tear. Despite 8 months of guided intensive rehabilitation, the subluxation persisted and maximal achieved knee flexion was 25°, with 10° of extension deficit. In February 2018, he was re-operated due to nonunion of the femoral shaft fracture. The stabilization hardware was removed, the nonunion was refreshed, and a Targon F nail (Aesculap) was introduced into the femur. A MUA of the knee performed during that procedure did not increase knee flexion and anterior tibial subluxation was still present (Fig. 1C). The surgeons at this point did not decide to perform any ligamentous release.

**Fig. 2:**
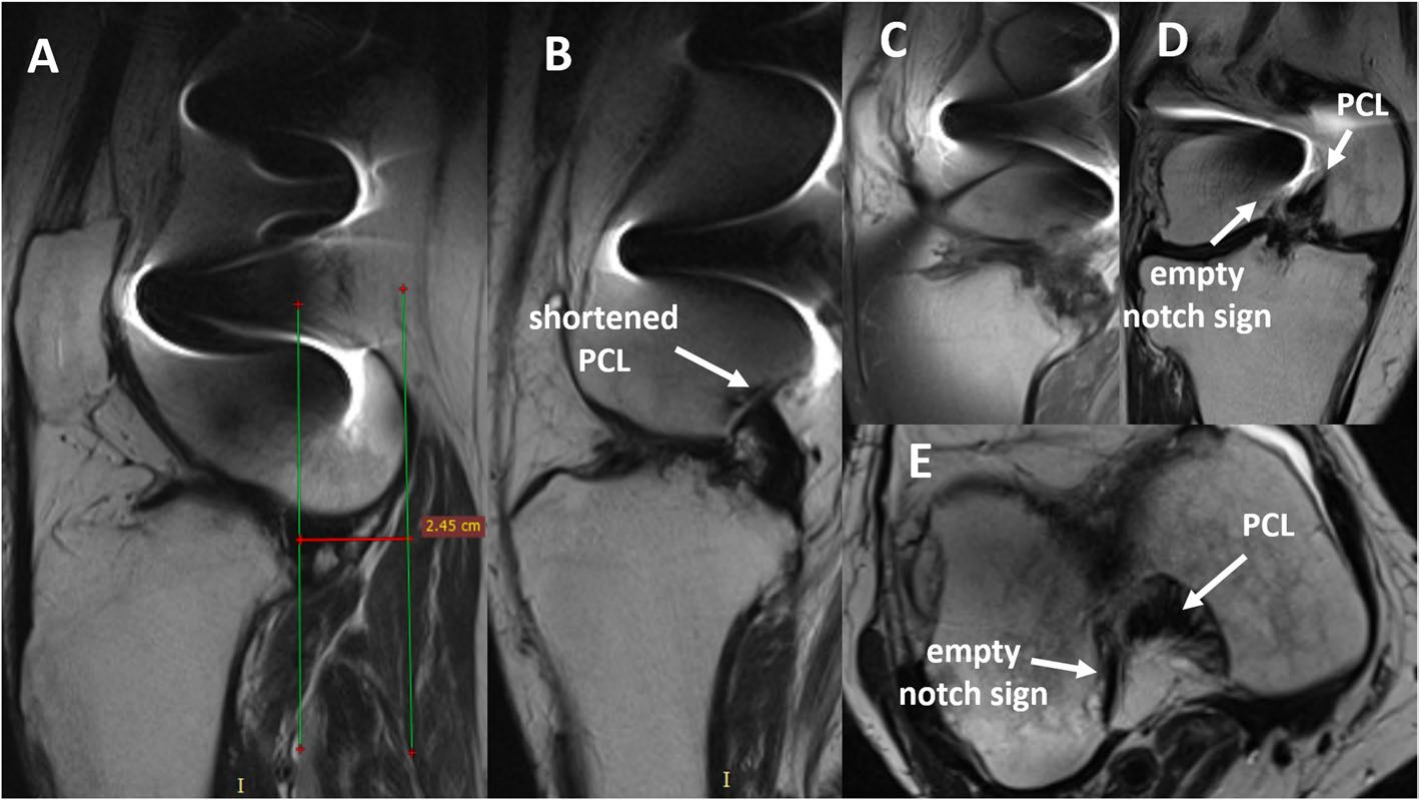
MRI at initial presentation to authors’ clinic. Anterior tibial subluxation (**A**) and PCL shortening (**B**) can be seen. No ACL fibers could be observed on sagittal (Fig. 2B, 2C), coronal (Fig. 2D) nor axial scans (Fig. 2E), in accordance with existing fixed anterior tibial knee subluxation. *ACL* Anterior cruciate ligament*, PCL* Posterior cruciate ligament

**Fig. 3:**
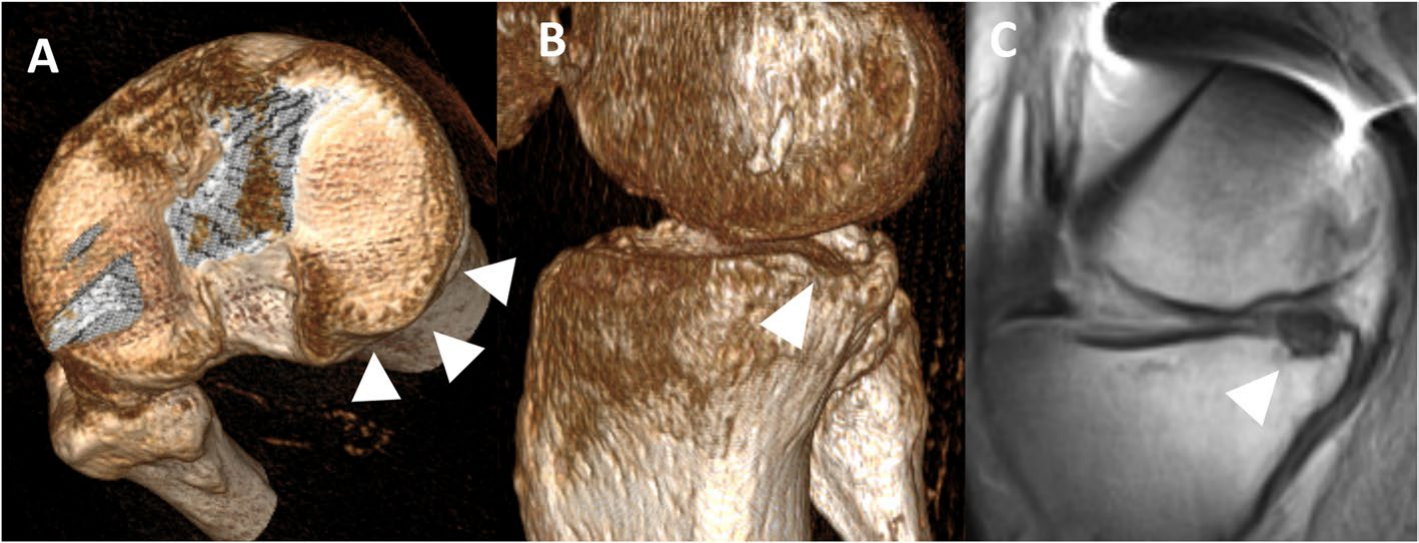
Depression fracture of posterior part of medial tibial condyle at initial presentation to authors’ clinic could be observed on CT (Fig. 3A, Fig. 3B) and MRI (Fig. 3C)

The patient was presented to the authors’ clinic in June 2019 with severe extension contracture (maximal flexion 25°, 10° of extension deficit), fibrotic and massively shortened quadriceps muscle, and fixed anterior tibial subluxation. On X-ray, fixed anterior tibial subluxation was confirmed (Fig. 1B-C) and clear signs of complex regional pain syndrome with increased local bone resorption could be seen (Fig. 1C). Magnetic resonance imaging (MRI) confirmed fixed anterior tibial subluxation (Fig. 2A). What is more, shortening of the posterior cruciate ligament (PCL) could be seen (Fig. 2B). No ACL fibers could be observed on sagittal (Fig. 2B, 2C), coronal (Fig. 2D), or axial MRI scans (Fig. 2E), in accordance with existing fixed anterior tibial subluxation. Furthermore, a depression fracture of the posterior part of medial tibial condyle could be observed on computed tomography (CT) and MRI (Fig. 3).

**Fig. 4:**
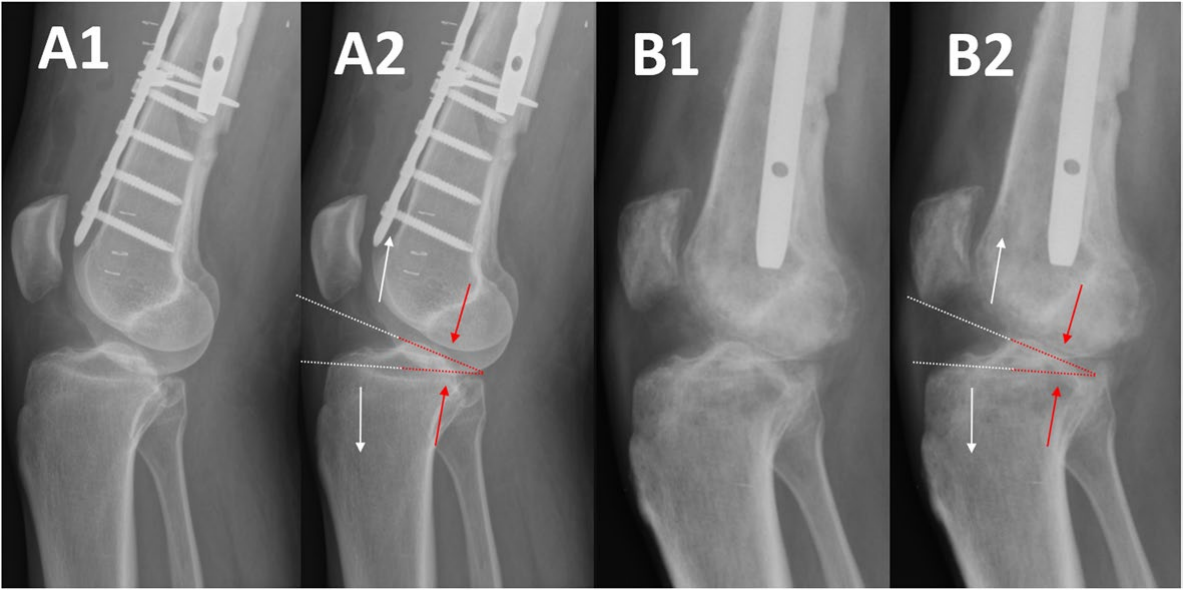
Fixed anterior tibial subluxation with incorrect joint congruence, resulting in abnormal “hinge-like movement” (A1,B1). Compression of posterior part of the knee and anterior joint opening could be seen during each attempt of knee flexion (A2,B2)

During each attempt of passive flexion, opening of the anterior knee was observed, accompanied with intense pain localized at the posterior aspect of the knee. The possibility to achieve 25° of flexion seemed to result from a “hinge-like” motion on the posterior rim of tibia instead of a natural gliding movement of the articular surfaces (Fig. 4).

In July 2019, the patient was operated on by the authors. During the first part of the procedure, extensive arthroscopic arthrolysis of the knee was performed, including anterior part of the knee, suprapatellar, medial and lateral recesses and the intercondylar notch. Due to the extension contracture, it was impossible to introduce the scope and instrumentation to the medial and lateral compartments, as well as the posterior recesses. Available to examination parts of the cartilage were graded maximally as ICRS Grade 2/3A [4].

**Fig. 5:**
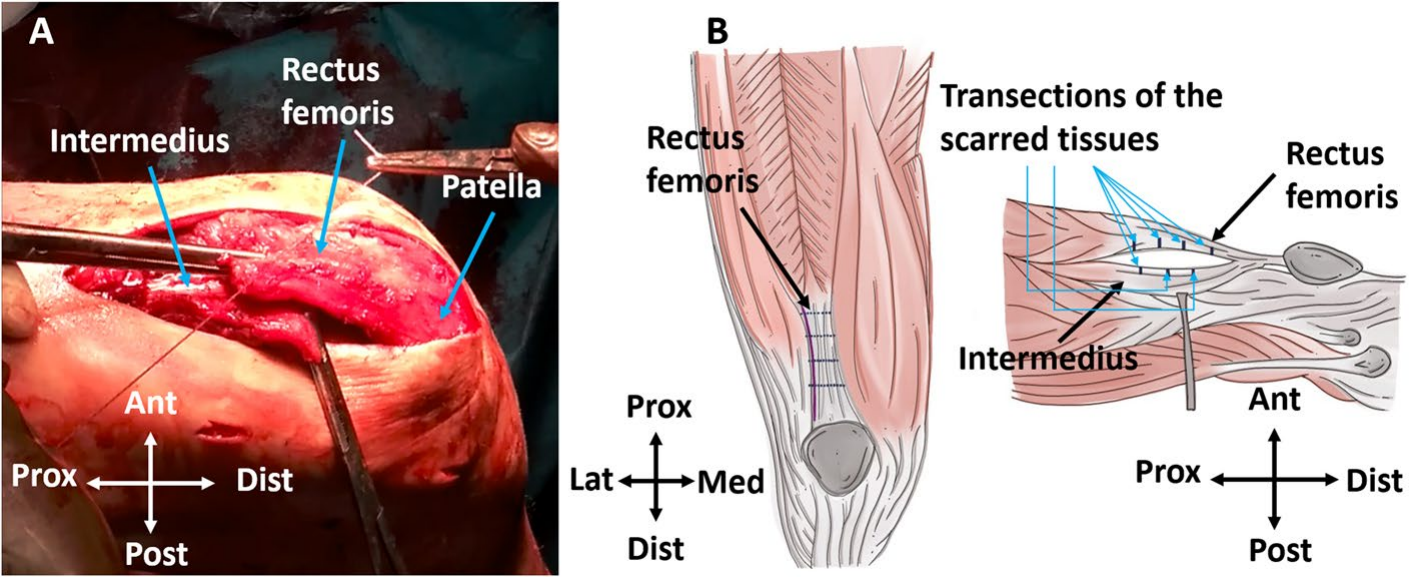
Two different quadricepsplasties techniques utilized during the first and second procedure in the authors’ clinic. **A** – Intraoperative photo of quadriceps Z-plasty with the intermedius transected near the patella and the rectus femoris about 7 cm proximal to the patellar base. **B** – Schematic drawing of open quadricepsplasty performed by making transverse of the fibrotic and scarred dorsal quadriceps tissues. *Prox* Proximal, *Dist* Distal, *Lat* Lateral, *Med* Medial, *Ant* Anterior, *Post* Posterior

Arthrolysis as described above followed by MUA did not increase knee flexion degree, however full knee extension was possible. Then, transverse quadriceps transections were performed in preparation for distal horizontal Z-quadricepsplasty, yet not sutured at this point. The vastus intermedius and rectus femoris aponeurotic part were horizontally dissected from each other. The intermedius was transected transversely near the patella and the rectus femoris about 7 cm proximal to the patellar base (Fig. 5A) [5]. We decided to perform this type of quadricepsplasty instead of i.e. Judet’s quadricepsplasty in order to limit the invasiveness of the procedure [2]. Lengthening of the quadriceps mechanism was achieved, however it only increased knee flexion to about 40°. What is more, the “hinge-like” movement (anterior knee opening, Fig. 4) was still present. Therefore, it was decided to release the shortened PCL at its femoral attachment. The release was achieved by transecting the PCL femoral attachment with the knife. Fibers were gradually transected and the possibility to translate the tibia into the physiological congruent position was tested. The anterior tibial subluxation was eliminated after transecting all PCL fibers. This maneuver restored more natural knee joint congruence and resulted in maximum flexion of 115°. The quadriceps parts were subsequently repaired with approximately 4 cm of elongation by suturing the transversely cut intermedius with transversely cut rectus femoris.

**Fig. 6:**
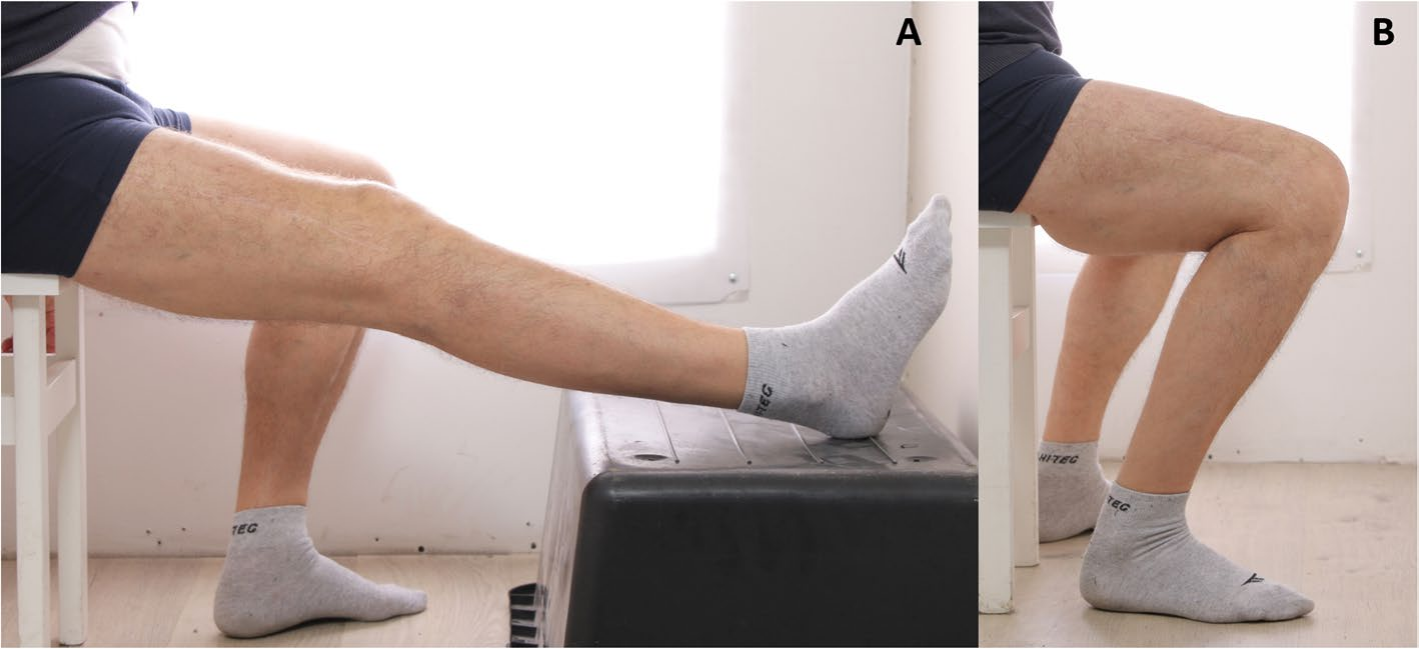
At 31 months follow-up, almost full knee extension (**A**) and 120° of knee flexion (**B**) were maintained

A supervised physiotherapy program was introduced. Weightbearing as tolerated, with crutches and straight knee tutor brace, was allowed from first postoperative day up to six weeks postoperatively. The brace was discontinued for sleeping and exercises. From the first postoperative day, the patient was demanded to perform 5–10 min sessions of passive knee flexion to at least 90 degrees and hyperextension every two hours. In the first three months postoperatively, the patient was undergoing weekly physical therapy focused on maintaining knee flexion and extension. Manual therapy of the postoperative scars and extension mechanism was performed to limit intramuscular adhesions formation. Restoration of muscular strength was gradually introduced in the following weeks. In the following months, supervised physiotherapy was performed at least every two weeks, accompanied with patient’s home exercises. Nevertheless, in a year the patient’s maximal flexion gradually decreased to 70° with remaining full extension and he was therefore advised to consider revision surgery. At the start of the procedure, revision arthroscopic arthrolysis was performed without significant increase of knee flexion. Therefore, open quadricepsplasty was performed more proximally than the previous one, by making transverse incisions of the fibrotic and scarred quadriceps tissues, similarly to the gastrocnemius lengthening technique summarized in the article of Moroni et al. (Fig. 5B) [6]. The longitudinal incision of the rectus femoris was performed along its lateral border (purple line on Fig. 5B). With iliotibial band and vastus lateralis retracted, the scarred quadriceps was transversely dissected on the thickest level in the localization corresponding to the space between rectus femoris and intermedius. Due to the previous quadricepsplasty, the tissues were scarred and fibrotic and therefore the dissection was performed in a mixed blunt and sharp manner. Afterwards, the transverse incisions of the fibrotic and scarred tissues were performed, aiming to transect one-third to one-half of the tissue thickness. As a last step of the procedure, MUA was performed. At this point, 120° of flexion was achieved intraoperatively. The second surgery was followed by the same physiotherapy protocol as the first. After six weeks, the patient was presented with 120° of knee flexion, 8° of active extension deficit, and 5° of passive extension deficit, which was maintained at final follow-up at 31 months postoperatively (Fig. 6). IKDC functional score improved from preoperative 4/87 to 49/87 at 12 months follow-up and 50/87 at 31 months follow-up. KOOS functional score improved from preoperative 7/100 to 69/100 at 12 months follow-up and 68/100 at 31 months follow-up. At the latest follow-up of 31 months, the knee was ACL- and PCL-deficient with antero-posterior instability assessed by the Lachman, posterior Lachman, anterior and posterior drawer tests (Grade 3 in each test). However, the rotational instability was more subtle (Grade 1 in the pivot-shift test, Grade 1 in the internal rotation dial test). Despite instability on clinical exam the patient was satisfied with the achieved improvement and did not want to undergo combined ACL and PCL reconstruction up to now. Furthermore, the intramedullary nail was not removed yet and at final follow-up there were still radiological and some clinical symptoms of residual algodystrophy. Therefore, the possible reconstructive procedure would require previous nail removal and further normalization of local tissue conditions.

**Fig. 7:**
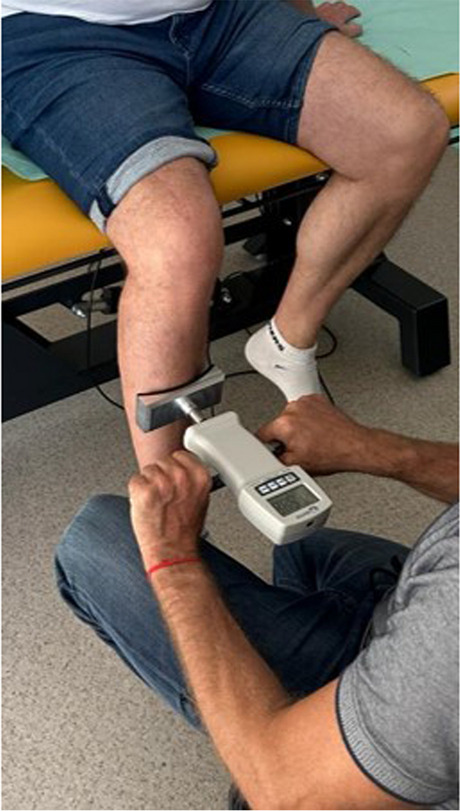
Assessment of quadriceps muscle strength by the means of FK1K electronic dynamometer. The knee was flexed to 90° and dynamometer was pressed in the middle of the shin

**Fig. 8:**
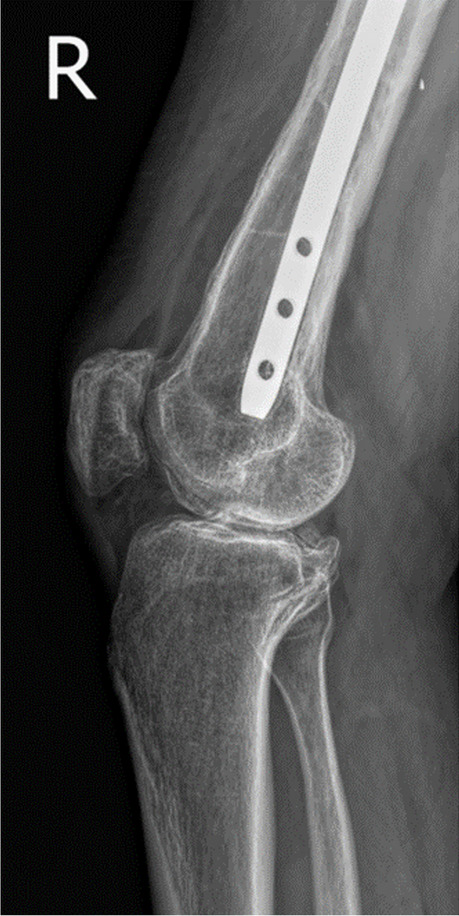
X-ray taken at 31 months follow-up

The quadriceps muscle strength after repeated quadricepsplasty was assessed at the 31-months follow-up by the means of FK1K electronic dynamometer (Sauter, Swiss), measurement resolution 0,5N, maximum force 1000N. The measurements were performed with knee flexed to 90 degrees with dynamometer in the middle of the shin (Fig. 7). Peak force was measured three times and the highest result was recorded. In the operated limb, 201N of extension force were achieved, compared to 227N on the contralateral side. Therefore it can be concluded that despite double repeated quadricepsplasty, the patient regained 89% of contralateral extension force in the operated limb, with 26N of difference. On X-ray taken at 31-months follow-up proper tibiofemoral joint congruence was present (Fig. 8).

## Discussion

There were two important findings of this study. First, PCL release in cases of severe extension contracture with fixed anterior subluxation allowed to achieve correct congruence of joint surfaces, and thus made subsequent knee flexion possible. Second, double repeated quadricepsplasty utilizing two different techniques was possible and effective despite massive fibrosis of the quadriceps muscle.

Release of PCL was reported in cruciate retaining total knee arthroplasty to avoid its excessive tension [7]; however, to the extent of the authors’ knowledge, it has not been well-described in cases of post-traumatic extension contracture. It remains to be studied what is the role of this procedure in the treatment of patients with fixed anterior tibial subluxation with incorrect congruence of joint surfaces. Obviously, such a procedure leads to ACL- and PCL-deficient knees and therefore should not be treated lightly. Nevertheless, the patient was satisfied with the achieved improvement and up to 31 months follow-up after the second procedure he did not want to undergo ligamentous reconstruction. As to quadricepsplasty, the authors are not aware of studies describing double repeated quadriceps-lengthening with use of two different techniques, as presented in this case report. The chosen quadricepsplasty techniques allowed us to limit the invasiveness of the procedures. It may be hypothesized that this is one of the reasons why good knee extension force was achieved at the final follow-up. No extensor mechanism rupture occurred and while there was a 8° extension lag (active extension deficit), functional outcomes were good.

The following conclusions could be drawn from this report. First: in cases of severe extension contracture with fixed anterior subluxation, subsequent PCL shortening may occur. Release of the PCL could allow to achieve correct congruence of joint surfaces, and thus make subsequent flexion possible. Second, double repeated quadricepsplasty utilizing two different techniques was safe and effective procedure, which suggests that it may potentially be utilized as a salvage procedure despite massive fibrosis of the quadriceps muscle.

## Data Availability

Not applicable.
